# Obesity Care in Primary Care: Implementing the GSA KAER Toolkit for Older Adults

**DOI:** 10.1093/geroni/igaf122.3983

**Published:** 2025-12-31

**Authors:** Anna Pendrey, Triccia Aparicio, Miguel Paz, Mariel Zelaya, Samir Cabrera, Javier Sevilla, Paola Rodriguez, Omar Suazo

**Affiliations:** Indiana University, Zionsville, Indiana, United States; Universidad Nacional Autonoma de Honduras, Zionsville, Indiana, United States; Universidad Nacional Autonoma de Honduras, Zionsville, Indiana, United States; Universidad Nacional Autonoma de Honduras, Zionsville, Indiana, United States; UNITEC, Zionsville, Indiana, United States; Indiana University, Zionsville, Indiana, United States; Indiana University, Zionsville, Indiana, United States; UNITEC, Zionsville, Indiana, United States

## Abstract

Background Obesity affects nearly 40% of US adults aged ≥65 years and contributes to cardiovascular disease, diabetes, functional decline, and reduced quality of life. Age-related physiological changes, multimorbidity, and polypharmacy complicate obesity management. The Gerontological Society of America’s KAER (Kickstart, Assess, Evaluate, Refer) Toolkit offers structured, age-appropriate guidance for primary care providers. Methods This quality improvement project was conducted in a geriatric primary care clinic from September 2023 to June 2025. The KAER Toolkit was used in routine visits for patients aged ≥65 years. Providers were trained to discuss weight without stigma, assess body composition and comorbidities, evaluate contributing factors, and refer patients. Data on screening, provider engagement, and referrals were analyzed. Results Among 41 older adult patients, 34% were identified with obesity, predominantly females aged 66–70 years. Most had class I obesity. Patients were diverse, with Hispanic (43%) and African American (36%) representation. Functional assessments showed that while most remained independent in basic activities of daily living, 71% required assistance with instrumental activities. High rates of depression (86%) and cognitive impairment (64%) were observed, along with prevalent comorbidities such as hypertension (93%), chronic kidney disease (86%), and hyperlipidemia (71%). Nutritional deficiencies were prevalent, with 64% deficient in vitamin D. Conclusions The GSA KAER Toolkit facilitated systematic, age-sensitive obesity management in primary care. Its user-friendly design supports early identification, targeted intervention, and coordinated care for obesity in older adults.

